# Metabolic effects of alternate-day fasting in males with obesity with or without type 2 diabetes

**DOI:** 10.3389/fphys.2022.1061063

**Published:** 2022-12-01

**Authors:** Arthur Ingersen, Hildegunn Rømma Helset, Monika Calov, Elizaveta Chabanova, Eva Gjerlevsen Harreskov, Christina Jensen, Christina Neigaard Hansen, Clara Prats, Jørn Wulff Helge, Steen Larsen, Flemming Dela

**Affiliations:** ^1^ Xlab, Center for Healthy Aging, Department of Biomedical Sciences, Faculty of Health and Medical Sciences, University of Copenhagen, Copenhagen, Denmark; ^2^ Department of Geriatrics, Bispebjerg-Frederiksberg University Hospital, Copenhagen, Denmark; ^3^ Department of Diagnostic Radiology, Copenhagen University Hospital, Copenhagen, Denmark; ^4^ Clinical Research Centre, Medical University of Bialystok, Bialystok, Poland

**Keywords:** fasting, glucose homeostasis, insulin sensitivity, visceral fat, weight loss, β-cell

## Abstract

Alternate-day fasting induces oscillations in energy stores. We hypothesized that repeated oscillations increases insulin secretion and sensitivity, and improve metabolic health in patients with obesity with or without type 2 diabetes (T2DM). Twenty-three male patients fasted every other day for 30 h for 6 weeks. Experiments included resting energy expenditure, continuous glucose monitoring, intravenous glucose tolerance test, euglycemic hyperinsulinemic clamp, body composition, hepatic triglyceride content, muscle biopsies which were performed at baseline, during 3 weeks without allowed weight loss, and after additional 3 weeks with weight loss. Bodyweight decreased ∼1% and further ∼3% during weeks one to three and four to six, respectively (*p* < 0.05). Only minor changes in fat mass occurred in weeks 1–3. With weight loss, visceral fat content decreased by 13 ± 3% and 12 ± 2% from baseline in patients with and without T2DM, respectively (*p* < 0.05). Hepatic triglyceride content decreased by 17 ± 9% and 36 ± 9% (with diabetes) and 27 ± 8% and 40 ± 8% (without diabetes) from baseline to week 3 and week 6, respectively (all *p* < 0.05). Muscle lipid and glycogen content oscillated with the intervention. Glucose homeostasis, insulin secretion and sensitivity was impaired in patients with T2DM and did not change without weight loss, but improved (*p* < 0.05) when alternate day fasting was combined with weight loss. In conclusion, alternate-day fasting is feasible in patients with obesity and T2DM, and decreases visceral fat and liver fat deposits. Energy store oscillations by alternate-day fasting do not improve insulin secretion or sensitivity *per se*.

**Clinical Trial registration:** (ClinicalTrials.gov), (ID NCT02420054).

## Introduction

Type 2 diabetes is a disease with increasing incidence that carries long-term complications and premature death. Key factors in the pathogenesis of type 2 diabetes are a combination of insulin resistance, insufficient insulin secretory capacity, and genetic disposition combined with excess energy intake and physical inactivity.

For patients with obesity and type 2 diabetes, increased physical activity and weight loss are the first-line of treatment. If unsuccessful, the treatment regimen proceeds with pharmacological therapy. The goal is to achieve good glycemic control and prevent long-term complications. To this end, all basic treatment regimens aim at improving insulin resistance and/or increasing insulin secretion. For lifestyle approaches, physical training (Dela et al., 1994; Dela et al., 1995; Holten et al., 2004) and weight loss, e.g. *via* gastric bypass surgery (Hansen et al., 2016), are effective in increasing insulin sensitivity. The effect of physical training on insulin secretion in patients with type 2 diabetes is less well studied, but a few studies have found that the insulin secretory capacity may increase (Krotkiewski et al., 1985; Dela et al., 2004; Solomon et al., 2010) following physical training. With weight loss by gastric bypass or diet, insulin secretory capacity increases in the sub-set of patients who have the shortest duration of the disease and the best preoperative β-cell function (Lund et al., 2015; Steven et al., 2016a). The mechanism for restoration of the insulin secretory capacity is believed to be a lessened gluco-lipotoxicity stress on the β-cells which will discontinue β-cell dedifferentiation (Poitout et al., 2010; White et al., 2016).

Overweight and patients with obesity often use specialized diet strategies as a means to reduce body weight and thereby improve glucose homeostasis. An increasingly popular diet is the intermittent fasting regimen, which includes alternate-day fasting (ADF) (Harvie and Howell, 2017), but the effect of ADF on weight loss is not better than everyday reduced caloric intake (Trepanowski et al., 2017; Carter et al., 2018). For normal-weight people or patients with obesity, a general health benefit of ADF with or without concomitant weight loss has not been established (Mattson et al., 2016; Trepanowski et al., 2017). Likewise, the effect of ADF on glucose homeostasis and diabetes risk indicators has been questioned (Heilbronn et al., 2005; Barnosky et al., 2014; Varady, 2016), although one study in young healthy males showed increases in insulin sensitivity following short term ADF (Halberg et al., 2005). Apart from an observational pilot study using a prolongation of overnight fasting that demonstrated a weight loss in consequence of reduced calorie intake but no change in insulin sensitivity (estimated by the homeostasis model assessment, HOMA + IR) (Arnason et al., 2017), studies on the effect of ADF on glucose homeostasis in patients with type 2 diabetes are sparse. However, one 12 months study in patients (N = 137) with type 2 diabetes has demonstrated that an intermittent energy restriction diet (500–600 kcal/day) on two separate weeks days resulted in similar positive effects on HbA1c levels and body composition as a regular continuous energy restriction diet (Carter et al., 2018).

In addition to the positive effect of weight loss, ADF may *per se* (i.e. without weight loss) have positive effects on glucose homeostasis in patients with type 2 diabetes. These patients are characterized by an elevated average blood glucose concentration, and therefore the β-cells are constantly exposed to a secretagogue. However, in consequence of zero caloric intake every other day, the secretory stimulus to the β-cells will be substantially reduced and therefore lessen the burden on the β-cells, which–in turn - may regain a higher secretory capacity. In addition, ADF may also reduce ectopic fat deposits in the pancreas and the liver (Kelley et al., 2003; Steven et al., 2016b) enabling a functional recovery of these organs (Al-Mrabeh et al., 2020).

Specific therapies aiming at reversing the β-cell dysfunction and improving insulin sensitivity in patients with type 2 diabetes are needed. Here we tested a diet regimen with 3 weeks of ADF without concomitant weight loss (i.e. on feast days the double amount of food were consumed) followed by 3 weeks with an *ad libitum* diet on feast days (i.e. a weight loss was allowed (ADF + WL)). We hypothesised that repeated oscillations in hepatic and intramuscular energy stores induced by ADF *per se*, would increase the β-cell secretory capacity and insulin sensitivity and improve glucose homeostasis in patients with type 2 diabetes. We expected that the additional weight loss with ADF + WL would improve β-cell secretory capacity and insulin sensitivity, hence these specific measures were demonstrated in a subset of the patients at the last follow-up. We further hypothesized that the diet regimen would decrease adipose tissue mass and liver fat content.

## Materials and methods

### Study participants

Twelve male patients with obesity and type 2 diabetes (T2DM) and eleven male patients with obesity (OB) aged 57 ± 6 and 55 ± 7 yrs (mean ± SD), and BMI 32.3 ± 2.6 and 32.9 ± 2.1 (kg/m^2^) respectively, were recruited *via* newspaper and website announcements. The time since diagnosis of type 2 diabetes was 2.3 ± 2.0 years. Patients with T2DM were in treatment with metformin (n = 8), metformin in combination with sulfonylurea (n = 1), metformin in combination with dipeptidyl peptidase-4 inhibitor (n = 1), angiotensin II-receptor blocker (ARB) (n = 2), ARB in combination with hydrochlorothiazide (n = 1), angiotensin-converting-enzyme inhibitor (ACE inhibitor) (n = 1), acetylsalicylic acid (n = 1), statin (n = 5) and ezetimibe (n = 1). The OB patients received ARB in combination with hydrochlorothiazide (n = 1), ARB (n = 1), and an ACE inhibitor (n = 1). All medication was discontinued from 1 week before the first baseline test through the 6 wk Intervention period. Exception from this was antihypertensive treatment, which was discontinued only on the test days and the day before.

Eligibility criteria were inactive males with obesity (BMI >28), either diagnosed with T2DM (Hba1c equal to or above 48 mmol/mol) or otherwise healthy. Absolute exclusion criteria were treatment with insulin and regularly performed sports activity more than once a week (assessed by interview). None of the participants performed regular sports activity on a weekly basis prior to inclusion and participants were asked to maintain their habitual activity level, during the intervention. Participants (T2DM and OB) were matched according to age and BMI. A flowchart of the recruitment process can be found in the supplemental material (Supplementary Figure S5). Experiments were performed at Xlab, Department of Biomedical Sciences at the University of Copenhagen. Magnetic resonance spectroscopies were performed at Department of Diagnostic Radiology, Copenhagen University Hospital Herlev-Gentofte.

### Experimental protocol

**FIGURE 1:**
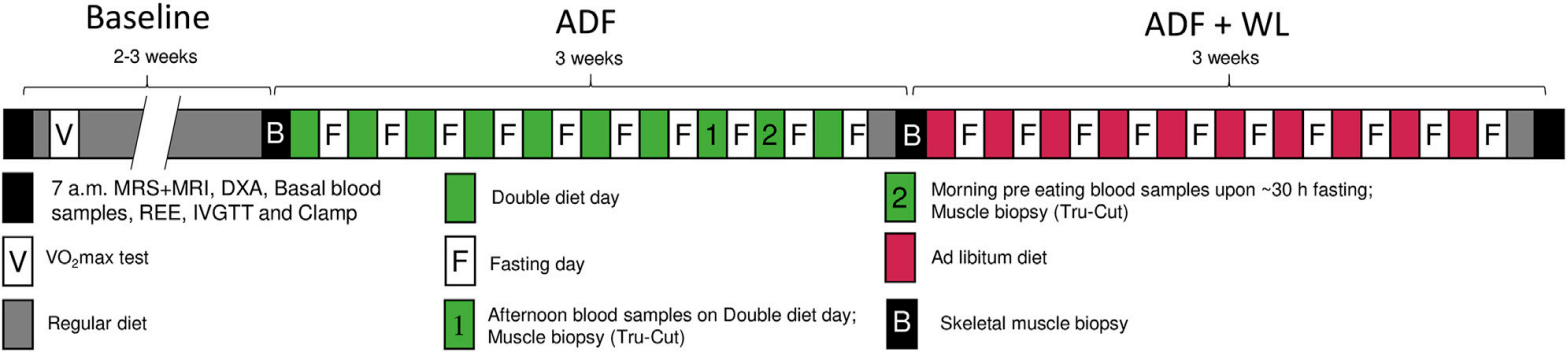
Study protocol. Two baseline experimental days were performed (black boxes) comprising of magnetic resonance spectroscopy (^1^H-MRS) and magnetic resonance imaging (MRI) (only one time at baseline), body composition (dual-energy X-ray absorptiometry (DXA) scan), fasting blood sampling, resting energy expenditure (REE), intravenous glucose tolerance test (IVGTT), and a euglycemic, hyperinsulinemic clamp These measurements were repeated after 3 weeks of alternate days fasting (ADF). During fast days (midnight continuing for 30 h until 06.00 the following day) zero calorie intake was allowed, and during the eating days, the participants ate the double amount of food. Bodyweight was measured every morning and the diet was adjusted accordingly so that no change in body weight should occur. Blood was sampled and Tru-Cut micro muscle biopsies were obtained on two occasions, one in the afternoon on a double diet day (marked 1), and one in the morning after fasting (marked 2). Subsequently. In the following 3 weeks, ADF was continued, however with no dietary restrictions, i.e. the study participants were allowed to lose bodyweight (ADF + WL). On the second baseline experimental day and following ADF (marked B), muscle biopsies were obtained before the IVGTT and after the clamp, in addition to the other procedures described. All tests were performed at the same time of day, at Baseline, ADF and at ADF + WL. Maximal oxygen uptake (VO_2_max) was measured at the study start to document a similar level of physical activity among all study participants.

Two baseline tests, separated by 2–3 weeks, were performed in the overnight fasting state and included resting energy expenditure by a ventilated hood (Jaeger Oxycon Pro, Intramedic, Hoechberg, Germany), body composition (dual-energy X-ray absorptiometry (DXA) scan (GE Medical Systems, Lunar iDXA Series, Madison, Wisconsin, United States)), a muscle biopsy from the vastus lateralis, blood sampling (arterialized blood by a heated hand vein), an 25 g intravenous glucose tolerance test (IVGTT) followed by a 2 h euglycemic (∼5.8 mM), hyperinsulinemic (insulin infusion rate: 80 mU/min/m^2^) clamp, that were concluded by a second muscle biopsy. The clamp included estimation of glucose kinetics by use of a stable isotope (6,6D_2_ glucose) infusion in a subgroup (T2DM, n = 6; OB: n = 8) during one of the two baseline clamps before and during the clamp after the first 3 weeks intervention (ADF). Plasma insulin concentrations during the clamp were increased to similar levels in T2DM and OB (1,406 ± 237 and 1,366 ± 188 pmol/l (Baseline); 1,343 ± 217 and 1,402 ± 288 pmol/l (ADF); 1,333 ± 260 and 1,199 ± 98 pmol/l (ADF + WL), respectively). The data from the two baseline tests did not differ significantly, and data are shown as pooled data. Between the two baseline tests, the per cent mean difference in measures of insulin sensitivity (glucose infusion rates, clamp data) were 2.2 ± 2.6 and 4.1 ± 4.1% in OB and T2DM, respectively. The second baseline test also included measurements of hepatic triglyceride content and skeletal muscle triglyceride content (m. psoas major) (by magnetic resonance spectroscopy (^1^H-MRS) and volume of visceral and subcutaneous fat (magnetic resonance imaging (MRI) at the level of L3. On a separate day, maximal oxygen uptake (VO_2_max) was determined by a graded bicycle exercise test until exhaustion (Jaeger Oxycon Pro, Intramedic, Hoechberg, Germany).Figure 1

Subsequently, an alternate day fasting (ADF) intervention was carried out for 6 weeks. The fasting day began at midnight (00:00 a.m.) and continued for 30 h until 06:00 a.m. the following day. During the fasting day only water, coffee and tea were allowed. During the first 3 weeks the participants aimed at maintaining their body weight by doubling their diet intake on the non-fasting days. No specific diet was prescribed, and the effect on body weight was monitored by daily weighing and reports to the investigators. The participants reported to the laboratory twice during the third week for blood sampling and a muscle biopsy (Tru-Cut needle), 1 day in the afternoon/evening on a double-diet day (i.e. non-fasting), and 1 day in the morning after ∼30 h fasting. After the 10th fasting day, a habitual eu-caloric diet for 1 day was followed by a repetition of the experimental procedures performed at baseline. The eu-caloric weight-maintaining diet was based upon the resting energy expenditure and the participant’s diet recordings during the 2–3 weeks between the two baseline tests. In the fourth, fifth, and sixth weeks of ADF, the participants followed an *ad libitum* diet on eating days, i.e. a weight loss was allowed. Then the baseline tests were repeated after 1 day of the habitual diet. Finally, continuous glucose monitoring (CGM) was performed by 7-day measurements of adipose tissue interstitial glucose concentrations (Medtronic iPro2 recorder, Medtronic A/S, Copenhagen, Denmark) in the baseline period, during ADF, and during ADF + WL.

This design aimed to focus on two different interventions, ADF without weight loss, i.e. the effect of ADF *per se*, and ADF with weight loss (ADF + WL), i.e. as the everyday practice of the method. All participants completed all tests and procedures, except that measures of insulin secretory capacity (IVGTT) and insulin sensitivity (clamp) were performed only in subgroups (T2DM: n = 6; OB: n = 2) on the final test day (ADF + WL).

Primary endpoints were changes in insulin sensitivity (by the hyperinsulinaemic euglycemic clamp, glucose clearance rate per fat-free mass) and beta-cell function (by IVGTT, insulin area under the curve) with ADF. Change in these with ADF + WL were exploratory. Changes in energy store levels were secondary outcome measures. These were prespecified as change in intramuscular glycogen content with fasting and double dieting, and change in intrahepatic and intramuscular triglyceride content with ADF and ADF + WL.

### Analyses

Plasma concentrations of substrates and metabolites (glucose, free fatty acids (FFA), glycerol, ß-hydroxybutyrate, cholesterol, triglyceride, high-sensitive C-reactive peptide (hsCRP), alanine aminotranferase (ALAT), asparatate aminotransferase (ASAT)) were measured by spectrophotometry (Cobas 6000 c 501, Roche, Glostrup, Denmark). HbA1c was analyzed on a DCA Vantage Analyser (Siemens Healthcare Diagnostics Inc. Tarrytown NY, United States). Concentrations of hormones in plasma were analyzed by ELISA technique: Insulin (Dako A/S, Glostrup, Denmark), C-peptide (80-CPTHU-E01; Alpco Diagnostics, Salem, HN, United States). Applied methods for resting energy expenditure, maximal oxygen uptake tests, whole-body dual-energy X-ray absorptiometry (DXA) scan (Hansen et al., 2015) and IVGTT (Dela and Stallknecht, 2010) have been described earlier. Methods for the measurement of hepatic triglyceride content (Chabanova et al., 2012), muscle triglyceride content (Fonvig et al., 2012) and volume estimation of visceral and subcutaneous fat have been described previously (Bille et al., 2012; Frossing et al., 2017). In brief, muscle and hepatic fat contents (%) were expressed as = $\frac{i_{0}\left(fat\right)}{i_{0}\left(fat\right)+i_{0}\left(water\right)}\times 100$ with $I_{\left(0\right)}$ as the initial areas of water and fat, respectively (Chabanova et al., 2012). Measurements were T2 corrected, and T2 was measured for each individual at each visit. Muscle intra- and extra myocellular content were not separated in the analysis, thus m. psoas major results are describing the sum of these. The 6,6D_2_ glucose isotope tracer/tracee ratio was determined by mass spectrometry (Sciex API 3000; Applied Biosystems, Foster City, CA). Protein content in m. vastus lateralis was examined using SDS-PAGE (Sodium Dodecyl Sulphate Polyacrylamide Gel Electrophoresis) and Western blotting techniques (see Supplementary Material). Analysis of mitochondrial function was performed in permeabilized skeletal muscle fibers. Mitochondrial respiratory capacity was determined as previously described (Dohlmann et al., 2018) using high-resolution respirometry (Oxygraph-2k, Oroboros Instruments, Innsbruck, Austria), and reactive oxygen species (ROS) production was measured fluorometrically (Xenius XC, SAFAS, Monaco) as previously described (Larsen et al., 2019) using the amplex red probe. See Supplementary Material for the detailed protocols. Citrate synthase (CS) activity was measured as previously described (Larsen et al., 2015).

Analysis of intramuscular triglyceride (IMTG) content was performed in muscle cryosections stained with Bodipy-493/503 as previously described (Prats et al., 2013). Image analysis was performed with Fiji software (Schindelin et al., 2012) to obtain lipid droplet size, density and IMTG fractional area in each cell. See Supplementary Material for the detailed protocols.

### Statistical analyses

Insulin sensitivity was estimated from glucose infusion rates during the final 30 min of the clamp. Glucose clearance rates were calculated as glucose infusion rates divided by the prevailing plasma glucose concentration. Glucose rate of appearance (Ra) was calculated using Steele’s steady-state equation (Steele et al., 1956).

All data are shown as mean ± standard deviation (SD).

Statistical analyses of parameters measured across the intervention phases (e.g. insulin secretion, insulin sensitivity, blood lipids, body weight) were performed with mixed model analyses (GraphPad Prism 8.0) This mixed model uses a compound symmetry covariance matrix and is fit using Restricted Maximum Likelihood (REML). In the absence of missing values, this method gives the same *p* values and multiple comparisons tests as repeated measures ANOVA. In the presence of missing values (missing completely at random), the results can be interpreted like repeated measures ANOVA. *p*-values < 0.05 was considered significant. Effectsizes were calculated using Cohen’s d. Based on data from previous studies (Halberg et al., 2005; Dela et al., 2019), we assumed that glucose infusion rates would increase 1.5 mg/min/kg and calculated that eight participants in each group (OB and T2DM) would provide an approximate power of 80%.

## Results

### Body composition

Maximal oxygen uptake was similar in the patients with type 2 diabetes (T2DM) and obesity (OB) (2.7 ± 0.1 and 2.9 ± 0.2 L per minute). Apart from the volume of visceral fat measured by MRI, the two groups were well matched concerning anthropometrics at baseline (Table 1). Bodyweight, body mass index (BMI), lean mass, fat mass, visceral and subcutaneous fat content all decreased significantly with ADF and ADF + WL (Table 1). However, the decrease was primarily due to the last part of the intervention protocol, where a weight loss was allowed. With ADF alone, bodyweight changed by ∼1% while a further ∼3% decrease was seen with ADF and allowed weight loss (AFD + WL) (Table 1). Lean mass changed minimally (between 1 and 3%), while total fat mass decreased by 5% with ADF + WL in both groups (Table 1). The most pronounced change in body composition (DXA scan) was the loss of visceral fat with ADF + WL, which decreased by 13 ± 3% and 12 ± 2% from baseline to ADF + WL in T2DM and OB, respectively (*p* < 0.0001) (Table 1). With MRI the volume of visceral fat decreased by 16 ± 3 and 12 ± 3% in T2DM and OB, respectively (*p* < 0.0001), and the volume was higher in T2DM compared with OB (Table 1 (*p* = 0.014)). ADF + WL lead to 7–8% subcutaneous fat loss (Table 1 (*p* = 0.0092)). Systolic and diastolic blood pressure decreased with the intervention (*p* = 0.0005 and *p* = 0.0018, respectively) and HbA1C decreased significantly in T2DM (Table 1 (*p* < 0.0001)). Basal metabolic rate, measured as resting energy expenditure, decreased throughout the intervention and thus reflected the weight loss (Table 1 (*p* = 0.0002)).

**TABLE 1:** Body composition and metabolic markers.

Table 1	Group	Baseline	ADF	ADF + WL	*p*-value		
					Group	Intervention	**Interaction**
Body mass (kg)	T2DM	103.6 ± 9.6	102.2 ± 9.5	99.6 ± 10.1	0.6348	**<0.0001**	0.2335
	OB	105.7 ± 9.9	104.6 ± 9.5	100.9 ± 8.8			
BMI (kg/m^2^)	T2DM	32.3 ± 2.6	31.8 ± 2.5	31.0 ± 2.7	0.5879	**<0.0001**	0.2466
	OB	32.9 ± 2.1	32.5 ± 2.0	31.4 ± 1.9			
Lean mass (kg) (DXA)	T2DM	64.6 ± 5.7	64.1 ± 5.5	63.3 ± 5.5	0.6400	**<0.0001**	0.0689
	OB	66.1 ± 6.8	65.6 ± 6.8	63.9 ± 6.7			
Fat mass (kg) (DXA)	T2DM	37.5 ± 6.9	36.4 ± 6.6	34.8 ± 6.8	0.9167	**<0.0001**	0.5045
	OB	37.6 ± 8.1	37.1 ± 8.7	35.1 ± 7.5			
Visceral fat (kg) (DXA)	T2DM	3.3 ± 0.7	3.0 ± 0.6	2.8 ± 0.6	0.0717	**<0.0001**	0.3382
	OB	2.7 ± 0.6	2.6 ± 0.6	2.3 ± 0.5			
Volume visceral fat (cm^3^) (DXA)	T2DM	3389 ± 703	3321 ± 657	2940 ± 680	0.0805	**<0.0001**	0.7512
	OB	2851 ± 661	2784 ± 680	2484 ± 537			
Volume visceral fat (cm^3^) (MRI)	T2DM	373 ± 63	354 ± 75	321 ± 90	**0.0138**	**<0.0001**	0.3537
	OB	299 ± 59	276 ± 52	264 ± 39			
Volume subcutaneous fat (cm^3^) (MRI)	T2DM	257 ± 97	252 ± 93	225 ± 93	0.3582	**0.0092**	0.2552
	OB	297 ± 101	285 ± 101	284 ± 107			
Systolic blood pressure (mmHg)	T2DM	139 ± 13	131 ± 16	128 ± 7	0.4324	**0.0005**	0.2364
	OB	132 ± 9	130 ± 12	125 ± 12			
Diastolic blood pressure (mmHg)	T2DM	86 ± 7	81 ± 9	78 ± 5	0.7356	**0.0018**	**0.0052**
	OB	83 ± 7	83 ± 8	83 ± 10			
Resting energy expenditure (kJ/day)	T2DM	7839 ± 951	7680 ± 971	7378 ± 741	0.1478	**0.0002**	0.3832
	OB	7502 ± 877	7554 ± 843	6716 ± 730			
HbA1c (mmol/mol)	T2DM	54 ± 8	53 ± 8	51 ± 7	**0.0195**	**<0.0001**	0.1524
	OB	37 ± 3	37 ± 3	37 ± 4			
Glucose infusion rate (mg/min/kg)	T2DM	5.1 ± 1.8	4.9 ± 1.9	6.9 ± 0.8	**0.0259**	**0.0050**	0.6591
	OB	6.7 ± 1.1	6.7 ± 1.3	8.6 ± 1.4			
Glucose clearance rate (ml/min/kg FFM)	T2DM	7.1 ± 2.6	6.6 ± 2.7	9.7 ± 1.1	**0.0125**	**0.0014**	0.4318
	OB	9.8 ± 1.7	9.7 ± 1.6	12.1 ± 1.5			

Table 1: Twelve males with obesity and type 2 diabetes (T2DM) and eleven males with obesity (OB) carried out 6 weeks of fasting every other day, i.e. from midnight and continued for 30 h until 06.00 the following day. The first 3 weeks of the alternate day fasting (ADF) the study participants had a double diet intake on eating days to avoid a change in body weight. During the following 3 weeks, there were no dietary restrictions, i.e. the study participants were allowed to lose body weight (ADF + WL). The volume of visceral and subcutaneous fat was measured with MRI (1 cm slice at the level of mid L3). Glucose infusion rates (GIR) and glucose clearance rates were derived from the final 30 min of a 120 min euglycemic, hyperinsulinemic (80 mu/min/m^2^) clamp performed at Baseline and at ADF (T2DM n, 11; OB n, 11), and in a subgroup at ADF + WL (T2DM n, 6; OB n, 2). The average of two baseline clamps performed 2–3 weeks apart is reported as the baseline value. *p*-values are derived from repeated measures ANOVA (or mixed model in case of missing data at random) and indicate the difference between groups (Group), the difference with the intervention across groups (Intervention) and group dependent effects of the intervention (Interaction), when *p* < 0.05. Data are shown as mean ± SD.

Bold values indicate *p* < 0.05.

### Biomarkers of the intervention

During the 6 weeks intervention, plasma glucose concentrations were always higher (Group: *p* < 0.0001; Intervention: *p* = 0.0003; Group x Intervention: *p* = 0.0020) in T2DM compared with OB (Table 2), and within T2DM glucose concentrations were highest and lowest after double diet and 30 h fasting, respectively (Table 2). Plasma concentrations of insulin and C-peptide were as expected elevated on the double diet day (Table 2). Insulin was not different between T2DM and OB, but C-peptide was higher in T2DM compared with OB (main effect, group: *p* = 0.0234; intervention: *p* < 0.0001; group x intervention: *p* = 0.0768). β-hydroxybutyrate (representing ketone bodies) were increased after 30 h Fasting, and higher in T2DM vs. OB (group: *p* = 0.0064; intervention: *p* < 0.0001; group x intervention: *p* = 0.0325) (Table 2). Plasma concentrations of adiponectin tended to be higher in OB compared with T2DM (group: *p* = 0.0643; intervention: *p* = 0.0079, group x intervention: *p* = 0.5700). Leptin was similar in T2DM and OB, but with no statistically significant interaction (group: *p* = 0.4112; intervention: *p* < 0.0001; group x intervention: *p* = 0.3158), the numerically lower values after 30 h fasting (Table 2) could not be statistically confirmed. However, testing the intervention effect separately in the two groups, revealed a significant (*p* < 0.05) reduced Leptin concentration after 30 h Fasting in both groups.

**TABLE 2:** Plasma markers of energy homeostasis and intramuscular glycogen content.

Table 2	Group	Baseline	Double diet	30 h fasting	ADF	ADF + WL
Glucose	T2DM	10.0 ± 2.2 †	11.8 ± 4.3 †*	8.2 ± 1.8 †*	9.6 ± 2.3 †	8.3 ± 1.9 †*
(mmol/l)	OB	5.9 ± 0.5	6.7 ± 0.7	6.0 ± 0.3	6.2 ± 0.6	5.8 ± 0.4
Insulin	T2DM	106 ± 49	380 ± 334 *	79 ± 28	119 ± 71	93 ± 37
(pmol/l)	OB	88 ± 48	476 ± 419 *	74 ± 41	102 ± 66	63 ± 30
C-peptide	T2DM	911 ± 316	2065 ± 539 *	694 ± 181	964 ± 396	797 ± 339
(pmol/l)	OB	654 ± 217	1,478 ± 471 *	557 ± 163	733 ± 313	587 ± 223
β-hydroxybutyrate	T2DM	0.08 ± 0.06	0.11 ± 0.08	0.30 ± 0.17 †*	0.07 ± 0.05	0.18 ± 0.19
(mmol/l)	OB	0.06 ± 0.04	0.03 ± 0.02	0.12 ± 0.05	0.05 ± 0.02	0.12 ± 0.08
Adiponectin	T2DM	1,151 ± 456	1,376 ± 566	1,320 ± 549	1,238 ± 534	1,261 ± 508
(ng/ml)	OB	1,591 ± 539	1,695 ± 530	1,741 ± 564	1,618 ± 461	1,637 ± 525
Leptin	T2DM	17.2 ± 12.9	13.8 ± 9.5	9.6 ± 8.7 *	16 ± 12.8	11.6 ± 9.5
(ng/ml)	OB	12.2 ± 5.8	13.2 ± 3.1	6.6 ± 2.8 *	12.3 ± 5.5	9.7 ± 7.7
Cholesterol	T2DM	4.9 ± 0.4	5.6 ± 0.7	5.1 ± 0.8	4.6 ± 0.8	4.5 ± 0.8
(mmol/l)	OB	5 ± 0.6	5 ± 0.8	5.2 ± 0.8	4.6 ± 0.7	4.4 ± 0.6
HDL-cholesterol	T2DM	1.0 ± 0.3	0.9 ± 0.3	1.1 ± 0.3	1.1 ± 0.3	1.1 ± 0.4
(mmol/l)	OB	1.1 ± 0.1	0.9 ± 0.1	1.1 ± 0.1	1.2 ± 0.2	1.1 ± 0.1
LDL-cholesterol	T2DM	3.4 ± 0.6	3.3 ± 1.1	3.5 ± 0.8	3.2 ± 0.8	3.1 ± 0.8
(mmol/l)	OB	3.5 ± 0.6	2.9 ± 0.7	3.2 ± 0.7	3.1 ± 0.6	3.0 ± 0.5
Triglyceride	T2DM	2.1 ± 0.9	4.0 ± 1.3 #	2.0 ± 0.6	1.3 ± 0.4 $	1.1 ± 0.4 $
(mmol/l)	OB	1.5 ± 0.3	3.5 ± 1.9 #	1.8 ± 0.5	1.2 ± 0.3 $	1.0 ± 0.2 $
Free fatty acids	T2DM	520 ± 156	453 ± 105	716 ± 200 #	472 ± 199	528 ± 152
(µmol/l)	OB	446 ± 74	389 ± 166	646 ± 185 #	339 ± 106	461 ± 134
Glycerol	T2DM	53.4 ± 2.4	63.7 ± 5.3	72.3 ± 8.7	55.0 ± 3.7	56.8 ± 3
(µmol/l)	OB	46.9 ± 2.8	62.3 ± 6.6	91.5 ± 10.4 #	46 ± 2.9	51 ± 3.1
Alanine aminotransferase	T2DM	33.2 ± 12.0	37.5 ± 16.4	35.8 ± 16.4	30.8 ± 12.6	24.1 ± 8.8 *
(U/l)	OB	26.3 ± 10.7	32.2 ± 19.6	30.9 ± 18.1	27.2 ± 11.3	20.1 ± 8.3 *
Asparatate aminotransferase	T2DM	25.2 ± 8.0	33.5 ± 15.8 *	33.0 ± 15.0 *	25.9 ± 11.1	22.4 ± 7.8 *
(U/l)	OB	24.7 ± 4.7	30.8 ± 10.5	29.7 ± 8.2 *	24.4 ± 3.9	22.8 ± 5.5 *
HsCRP	T2DM	2.83 ± 3.08	2.05 ± 1.48	2.02 ± 1.35	2.31 ± 2.1	1.9 ± 1.47
(mg/l)	OB	3.02 ± 3.27	4.99 ± 4.72	3.61 ± 2.15	2.14 ± 1.63	1.92 ± 1.46
Glycogen	T2DM	404 ± 88	351 ± 79	307 ± 53 §	351 ± 70	-
(nmol/mg dry weight, muscle)	OB	412 ± 88	396 ± 91	350 ± 129 §	412 ± 110	-

Table 2: Plasma markers of energy homeostasis and intramuscular glycogen content in twelve patients with obesity and type 2 diabetes (T2DM) and eleven patients with obesity (OB) who participated in a 6 weeks alternate day fasting (ADF) protocol (see Figure 1). Venous blood samples were obtained in the fasting state at study start (baseline), in the afternoon/evening on a double-diet day (i.e. non-fasting), after 30 h of fasting, in the morning in the fasting state after 3 weeks with alternate-day fasting without weight loss (ADF), and in the morning in the fasting state after 3 weeks with alternate-day fasting with allowed weight loss (ADF + WL). Glycogen content in skeletal muscle were obtained *via* microbiopsies (Tru-Cut) in the evening after a day with a double diet and after 30 h fasting.

Data are mean ± SD. * denotes different from baseline within the group, # denotes different from all other time points within the group, $ denotes different from baseline, † denotes different from OB, within the time-point, § denotes different (*p* = 0.0308) from double diet across the groups (OB + T2DM, pooled). Post-hoc test: Sidák’s multiple comparisons test (between group) and Tukey’s multiple comparison test (within group).

Total cholesterol, LDL, and HDL cholesterol were not different between the groups and did not change with the intervention (Table 2). However, triglyceride concentrations in plasma increased in both groups with double diet (Table 2) and decreased with ADF (T2DM: −33 ± 6%; OB: −23 ± 4%) and ADF + WL (T2DM: −43 ± 5%; OB: −29 ± 4%) compared with baseline (Table 2). Plasma FFA concentrations were higher in T2DM compared with OB (main effect, group: *p* = 0.0408; intervention: *p* < 0.0001; group x intervention: *p* = 0.09539) (Table 2), and FFA concentration were increased in both groups following 30 h fasting. Plasma glycerol concentrations displayed a similar pattern, however, with no group differences, and only OB displayed a significant increase with fasting (Table 2). Notably, compared with baseline plasma triglyceride significantly decreased with ADF and ADF + WL (Table 2).

Hepatic biomarkers showed a slight decrease in ALAT and ASAT with ADF + WL, and ASAT was elevated with double diet and 30 h Fasting, but no differences between the groups were seen (Table 2). Plasma hsCRP values were generally very low, and no changes were seen (Table 2).

### Insulin secretion and sensitivity; muscle glycogen

**FIGURE 2:**
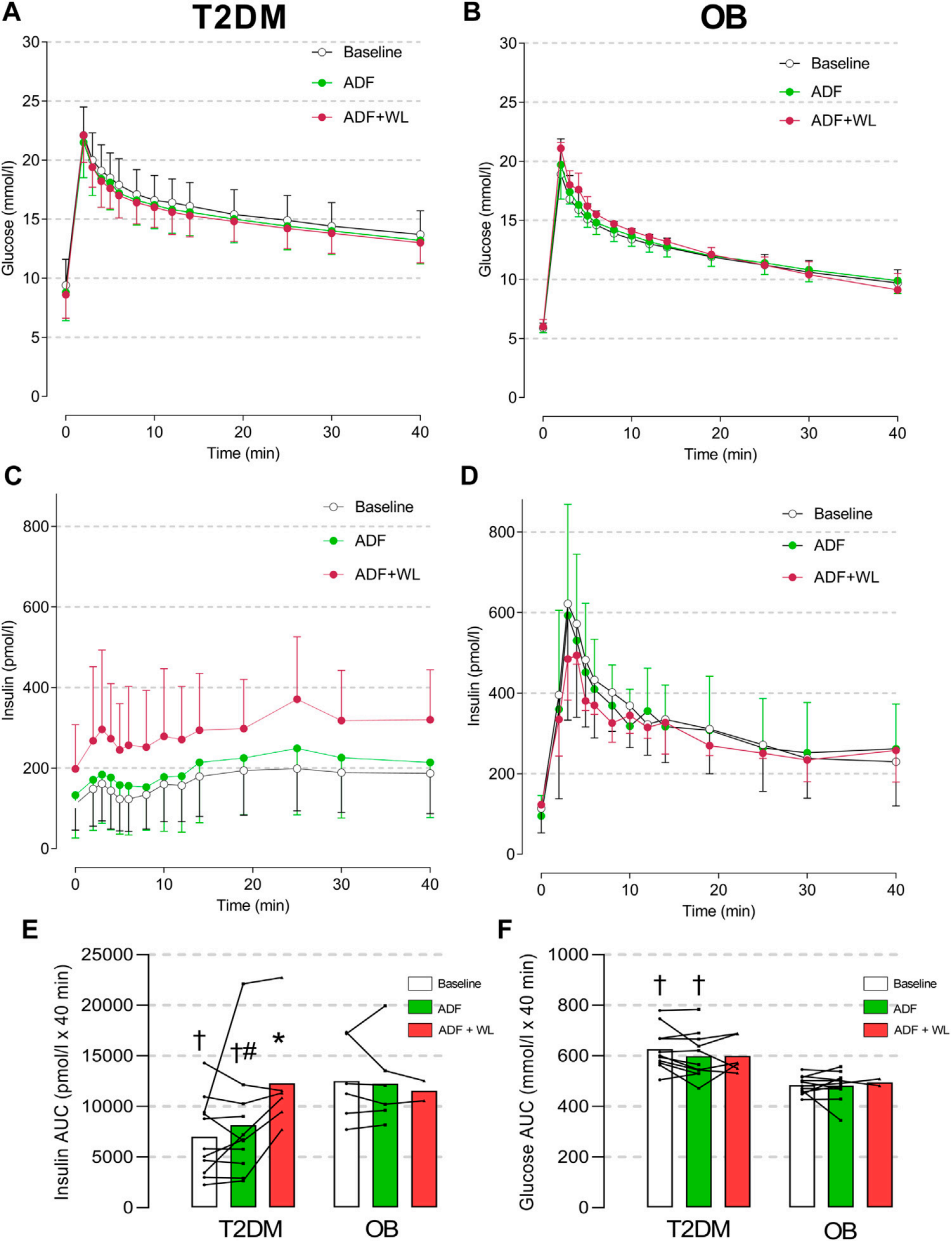
Plasma concentrations of glucose **(A and B)** and insulin **(C and D)** in response to an intravenous glucose tolerance test (IVGTT) in patients with obesity (OB; **(B and D)** and type 2 diabetes (T2DM; **(A and C)** The participants were studied at baseline (mean of two baseline test are shown; T2DM n = 11, OB n = 11), after 3 weeks of alternate-day fasting (ADF) with double diet on non-fasting days (T2DM n = 11, OB n = 6) and after further 3 weeks ADF with *ad libitum* diet (i.e. weight loss was allowed (ADF + WL); T2DM n = 6, OB n = 2)) (see Figure one). In column graphs are shown area under the curve (AUC) for insulin **(E)** and glucose **(F)**. Data are shown as mean ± SD. Insulin AUC: Group (*p* < 0.0001), Intervention (*p* = 0.0007), Group x Intervention (*p* = 0.0001). # denotes different from ADF + WL, * denotes different from baseline within the group, and † denotes different from OB.

Intravenous glucose tolerance did not change with ADF or ADF + WL and was better in OB (Figure 2). The insulin response to the IVGTT was always lower in T2DM compared with OB (*p* < 0.0001), and the first phase insulin response was characteristically diminished in T2DM compared with OB. The interventions did not change the insulin response in the control group. ADF did not change insulin response in the patients with type 2 diabetes, but the subset of patients that had an IVGTT perfomed after ADF + WL (T2DM, n = 6) displayed improved (*p* < 0.0001) insulin response, compared with baseline and with ADF (Figure 2C). Thus, in the T2DM group, the average insulin concentration during the IVGTT was 162 ± 27 pmol/L at baseline, while it was 288 ± 58 pmol/L after ADF + WL (*p* < 0.05) (Figure 2E). The insulin response in OB at ADF + WL is merely displayed (Figure 2D) for illustrative purposes, and not subject to statistical analysis (n = 2). The area under the curve (AUC) for insulin in T2DM increased with the intervention (*p* = 0.0007) and there was a group × intervention interaction (*p* = 0.0001). Post-hoc test revealed Insulin AUC was lower in T2DM compared with OB at baseline and after ADF, and that insulin AUC increased from ADF to ADF + WL in T2DM, where insulin AUC was higher at ADF + WL compared with baseline (Figures 2E, F).

T2DM patients displayed insulin resistance (glucose infusion rate) compared with OB (Table 1). There was a significant effect of the intervention (*p* = 0.005), a difference between groups (*p* = 0.0259), but no interaction between the two (*p* = 0.6586), i.e. the insulin sensitivity increased as a main effect and not with ADF alone. With the accompanying weight loss (AFD + WL) insulin sensitivity increased in all subjects (Table 1), whether data are given as glucose infusion rates per min per kg body weight, or expressed as glucose clearance rates per kg fat-free mass (FFM) a similar pattern is seen (Table 1).

Glucose rate of appearance (Ra) after an overnight fast in the baseline test was increased in T2DM compared with OB (3.7 ± 0.2 vs. 3.1 ± 0.1 mg/min/kg, respectively (*p* < 0.05)), and decreased (*p* < 0.05) in both groups with insulin infusion during the clamp (to -0.1 ± 0.2 and 0.2 ± 0.2 mg/min/kg, respectively (*p* > 0.05)). Glucose Ra was unchanged in the fasting state following 3 weeks of ADF (T2DM: 3.8 ± 0.2; OB: 3.0 ± 0.1 (*p* < 0.05), and decreased with insulin (data not shown).

Skeletal muscle glycogen content was similar on clamps days in T2DM and OB (404 ± 88 and 412 ± 88 nmol/mg dry weight, respectively) and it was not notably changed after 3 weeks ADF (351 ± 70 and 412 ± 110 nmol/mg dry weight, respectively). No change in glycogen content could be detected with the hyperinsulinemic clamp in T2DM (Baseline clamp: Δ 54 ± 85; ADF clamp: Δ -76 ± 70 nmol/mg dry weight) or in OB (Baseline clamp: Δ −24 ± 92; ADF clamp: Δ -34 ± 80 nmol/mg dry weight).

Muscle glycogen content (Table 2) was also measured in the evening after a double diet day and in the morning after a fasting day (after 30 h fasting) (see protocol, Figure 1). In the pooled data set a significant (*p* = 0.0308) difference was seen, with glycogen content being lower after 30 h fasting. No significant difference was seen with the analysis of the OB and T2DM group separately.

### Continuous glucose monitoring

**FIGURE 3:**
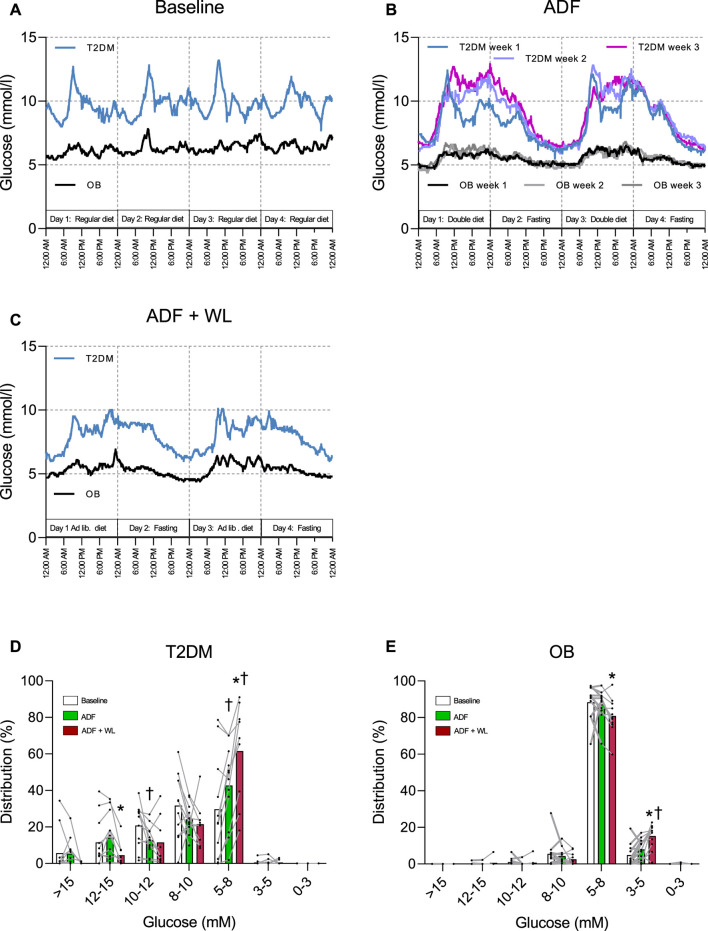
Continuous glucose monitoring (CGM) traces in patients with obesity (OB) and type 2 diabetes (T2DM). Data are shown as mean values. **(A)** four consecutive days during 1 week between baseline tests; **(B)** four consecutive days during week 1, 2 and 3 of alternate day fasting (ADF); **(C)** four consecutive days during 1 week with allowed weight-loss (ADF + WL). The distribution of time (%) (mean ± SD) spent in ranges of glycemia are shown in column graphs for patients with T2DM **(D)** and OB **(E)** during baseline, ADF and ADF + WL. † denotes different from Baseline, * denotes different from ADF.

Continuous glucose monitoring (CGM) was performed over four consecutive days in 1 week at baseline and during ADF + WL, but during ADF CGM was performed in all 3 weeks (each week on four consecutive days (Figure 3). The purpose was to monitor adherence to the fasting, double diet and *ad libitum* diet days and all subjects followed the intervention. Glucose levels were higher in T2DM compared with OB at all times (Figure 3). During ADF, CGM data from weeks one to three illustrates that eating on double diet days increased after the first week, probably also due to encouragement to the patients based upon the daily home-based weighing. CGM data are summarized in Figures 3D, E, showing that with ADF and ADF + WL the time spent in the lower ranges of glycemia increased with the intervention and this occurs in both groups. No hypoglycemic events were recorded.

### Hepatic and skeletal muscle lipids

**FIGURE 4:**
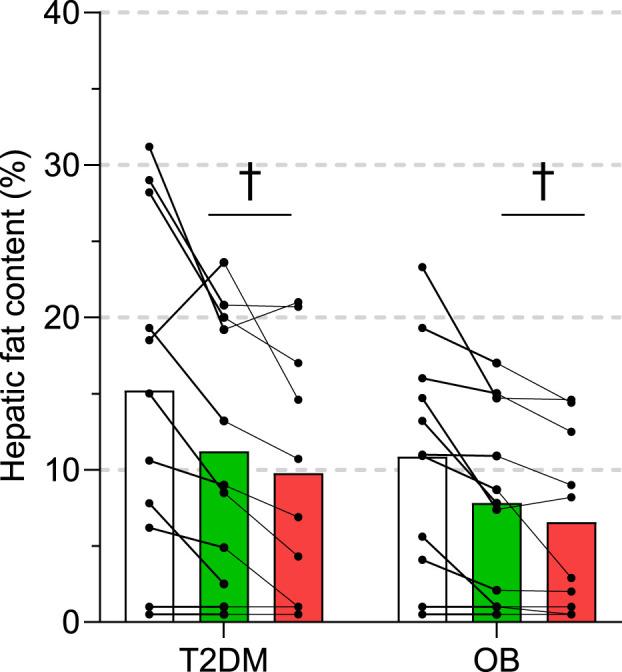
Intrahepatic triglyceride content measured by ^1^H-magnetic resonance spectroscopy in eleven subjects with obesity (OB) and eleven patients with type 2 diabetes (T2DM). Experiments were performed at baseline, after 3 weeks of alternate-day fasting with double diet on non-fasting days (ADF), and after 3 weeks of alternate-day fasting with *ad libitum* diet, i.e. weight loss was allowed (ADF + WL). Data are shown as mean ± SD. † denotes different from baseline (*p* < 0.05).

Intrahepatic triglyceride content (by ^1^H-MRS) was at baseline 15 ± 11% (range: 1—31%) and 11 ± 7 (range: 1—23%) in T2DM and OB, respectively, and not different between the two groups throughout the intervention (Figure 4). With both ADF and ADF + WL a significant decrease from baseline was seen (Figure 4), but with considerable individual variation. Thus, ADF resulted in a 22 ± 25% (T2DM) and 27 ± 27% (OB) decrease, and ADF + WL resulted in a 36 ± 27% (T2DM) and 40 ± 25% (OB). The decrease in intrahepatic triglyceride content correlated significantly (*p* = 0.0005; *R* ^2^ = 0.57) with the increase in insulin secretion (Figure 2E) from baseline to ADF + WL.

Triglyceride content in m. psoas major (by ^1^H-MRS) was not different between the groups and did not change with the intervention (baseline: 5.6 ± 3.6% and 4.8 ± 4.0%; ADF: 5.8 ± 2.7% and 3.8 ± 3.3%; ADF + WL: 6.5 ± 3.1% and 4.4 ± 4.1%, in T2DM and OB, respectively).

**FIGURE 5:**
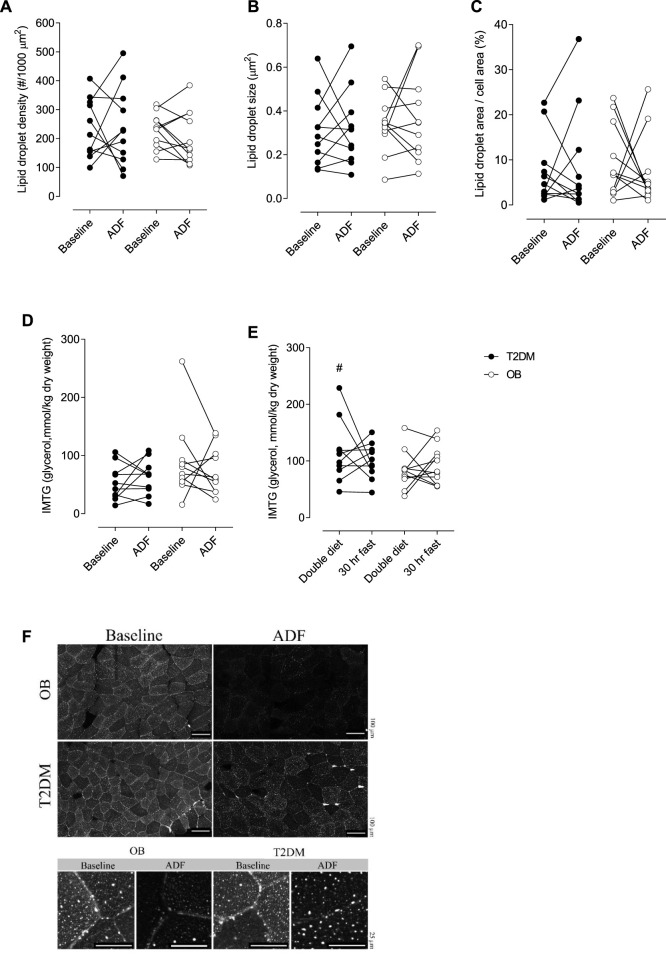
Lipid droplets (LD) density per area of muscle fibre **(A)**. LD size **(B)**. Fractional area of LDs’ **(C)**. Intramuscular triglyceride (IMTG) content in muscle at baseline clamp and at the clamp after ADF **(D)**. IMTG content in muscle after one double diet day and after 30 h fasting **(E)**. Representative images of Bodipy staining **(F)**. OB, Obese subjects, T2DM, patients with type 2 diabetes. In figure f, scale bars represent 100 µm (top) and 25 µm (bottom). # denotes different from baseline in Figure 4E (*p* = 0.0093).

Muscle lipid droplet density, size of droplets, or the fractional area of the droplets did not differ between T2DM and OB, and no change with ADF was seen (Figure 5A, B, C, F). These data were confirmed by analysis of intramuscular triglyceride (IMTG) content (Figure 5D). In addition, IMTG content was also measured in Tru-Cut biopsies obtained in the evening after a double diet day and in the morgen after 30 h fasting (Figure 5E). In T2DM, IMTG was significantly higher (*p* = 0.0093) after a double diet day compared with IMTG content on the clamp day (i.e. after an overnight fast), but this could not be detected in OB (Figure 5E). No difference in IMTG between double diet day and 30 h fasting could be seen in either group (Figure 5E).

### Skeletal muscle protein expression

The expression of proteins relevant for insulin-mediated glucose metabolism is shown in Supplementary Figure S1A-I. Proteins involved in glucose transport (GLUT4) and glycolysis (hexokinase and pyruvate kinase) and glycogen storage (glycogen synthase and phosphorylase) were similar between the two groups and did not change with ADF. Proteins relevant for GLUT4 vesicle formation showed minor changes. Thus, Akt was significantly higher in OB compared with T2DM, and AMPKα1 increased significantly with ADF in OB, but not in T2DM. AS160 and PKCθ remained unchanged and similar in the two groups.

The expression of proteins relevant to lipid metabolism is shown in Supplementary Figure S1J-S. Proteins involved in fatty acids transport into muscle cells were studied by analysis of proteins located in the plasma membrane. This was carried out to determine if alternate day fasting had any effect on fatty acid transport in healthy or diabetic skeletal muscle tissue. Fatty acid translocase (FAT) or CD36 binds long-chain fatty acids and is a key player in fatty acid transport across the plasma membrane. In the control group, the protein content of CD36 increased by 14% (*p* = 0.0424) after the intervention (Supplementary Figure S1J). The effect size was moderate (d = 0.53). Fatty acid transport protein 4 (FATP4) esterifies long-chain fatty acids and has a role in fatty acid transport across the plasma membrane. No change in FATP4 was observed in the control group and a decrease (*p* = 0.0066) was seen in the T2DM group (Supplementary Figure S1K). Plasma membrane fatty acid binding protein (FABPpm) is also involved in myocellular uptake of long-chain fatty acids. No change in FABPpm was observed in the control group but a tendency towards a decrease of 14% (*p* = 0.094) was found in the T2DM group after the intervention (Supplementary Figure S1I). The effect was of moderate (d = 0.47) size. No change was observed in the T2DM group or between the two groups for all fatty acid transporters.

### CS activity and mitochondrial function in skeletal muscle

Citrate synthase (CS) activity was used as an index for mitochondrial mass (Larsen et al., 2012). CS did not change from baseline to ADF and was similar between T2DM and OB (115 ± 26 to 101 ± 29 and 127 ± 42 to 117 ± 35 μmol/g/min, respectively). Oxygen consumption *ex vivo* was measured with a sequential substrate protocol, with state 2 respiration (complex I; malate and glutamate) followed by state 3 respiration with increasing concentrations of ADP (complex I) and dual electron input to complex I and II (glutamate, malate, succinate, and ADP), ending with uncoupled respiration (FCCP as protonophore) (Supplementary Figure S2A). Cytochrome c was added as quality control (max 10% increase in oxygen flux). First of all, there were no differences in respiration between T2DM and OB, and no significant effect of ADF was observed (Supplementary Figure S2A). Reactive oxygen species (ROS) displayed no difference between the groups and no effect of ADF was observed (Supplementary Figure S2B). ADP sensitivity and maximal oxygen flux (Vmax) was calculated from the oxygen flux during increasing ADP concentrations, and no differences between groups or effects of ADF were seen (Supplementary Figures S2C, D).

## Discussion

The present study represents a comprehensive characterization of the effects of alternate-day fasting regimens on the human metabolism, studied in obese patients with and without type 2 diabetes. The energy balance is essential in every attempt to lose bodyweight, and if weight loss is the primary focus it is fundamental to achieve a negative energy balance, no matter how this is brought about. This was not the primary focus here. With the present study protocol, we aimed to study the metabolic effects of oscillations in energy intake and thus energy balance.

An improvement of the insulin secretory capacity in patients with type 2 diabetes is a therapeutic goal that is difficult to achieve non-pharmacologically, and only a few studies in patients with type 2 diabetes have reported improvements in β-cell secretion following physical training (Krotkiewski et al., 1985; Dela et al., 2004; Solomon et al., 2010). The purpose of the present study was to mimic the oscillations that occur in energy stores with frequent exercise training, but at the same time avoid the physiological impact on metabolism that takes place with exercise training (i.e. muscle contractions). ADF would largely accomplish this, but from previous studies, it is known that weight loss often follows ADF. Therefore, we divided the study into two 3-week periods of ADF, where weight loss was allowed in the latter period, as would be the every day practice.

A large weight loss (14%–30%) achieved through gastric bypass surgery is known to improve insulin secretory capacity in patients with type 2 diabetes (Lund et al., 2015; Steven et al., 2016b), but to our knowledge, this is the first study to demonstrate that even a small weight loss achieved *via* ADF may have the same effect. We tested the hypothesis that alternate-day fasting, without concomitant weight loss, would increase the β-cell secretory capacity in patients with type 2 diabetes, and the data show that ADF alone (albeit with a ∼1% weight loss) does not significantly change insulin secretion in response to an IVGTT. However, the decrease in intrahepatic triglyceride content correlated significantly (*p* = 0.0005; *R* ^2^ = 0.57) with the increase in insulin secretion (Figure 2E) from baseline to ADF + WL. In the part of the experiment with allowed weight loss (ADF + WL), which included a ∼4% loss of body weight, ∼13% loss of visceral fat, and 36 ± 27% (range: 0%−84%) loss of intrahepatic triglyceride content, a significant increase in insulin secretion (compared with baseline) was demonstrated in a subgroup of patients with type 2 diabetes. The first-phase insulin response in the patients with type 2 diabetes was, however, not restored albeit the insulin response curve displayed a more marked first phase profile compared with baseline (Figure 2C). An indication that time-restricted feeding (i.e. not the same protocol as used in the present study) without weight loss may increase ß-cell responsiveness in pre-diabetic people has been published (Sutton et al., 2018), but this was shown with an oral glucose tolerance test, which is a non-steady test not well suited for accurate measurements of insulin secretion or insulin sensitivity.

The mechanism for improvements in insulin secretion has been attributed to a decrease in intrapancreatic triacylglycerol (Lim et al., 2011; Steven et al., 2016b). This is also a likely explanation in the present study, where we observed large decreases in visceral fat (Table 1) and intrahepatic triglyceride content (Figure 4) where the latter correlated significantly with the improvement in insulin secretion. The elevated plasma concentrations of FFA, glycerol, and β-hydroxybutyrate during fasting (Table 2) testified to an increased lipolytic rate during fasting, contributing to the marked decrease of adipose tissue during the interventions (Table 1). The decrease in insulin concentrations during each fasting period (Table 2) would also contribute to an increased lipolytic rate, and in particular with ADF + WL during which there were no excess calorie intake. An additional mechanism for the improvement in insulin secretion could also be due to an overall reduced glycemic load on the β-cells (i.e. reduced glucotoxicity). Apart from documenting the adherence to the protocol, the continuous glucose monitoring (Figure 3) revealed a lessened glycemic burden, which in itself reduces the stress on the β-cells.

It is important to note that the duration of type 2 diabetes, or at least the time since diagnosis, was short among the included patients (2.3 ± 2.0 yrs; range: 3 months—7 yrs). This means that the patients had a relatively well-preserved β-cell function, but of course, diminished compared with the obese subjects without type 2 diabetes (Figure 2). We have previously shown that patients with a high pre-operative β-cell function experience a superior outcome to gastric bypass surgery compared with those patients with the lowest pre-operative β-cell function (Lund et al., 2015) and that only such patients benefit from physical training in terms of β-cell function (Dela et al., 2004). Most likely, patients with severely reduced insulin secretory capacity (which can be easily estimated by a 6 min glucagon test (Dela et al., 2004)) will not benefit from ADF interventions in terms of insulin secretory capacity.

Insulin resistance was present in the patients with type 2 diabetes, as evidenced by the clamp data (table 1) and supported by the presence of hyperglycemia (Table 2; Figure 3), elevated HbA1c (Table 1), and a tendency to reduced adiponectin concentrations (main effect, *p* = 0.0643) in T2DM compared with OB (Table 2). Many studies have shown positive effects of training on insulin-mediated glucose uptake in skeletal muscle in patients with type 2 diabetes (Dela et al., 1995; Holten et al., 2004; Dela et al., 2019), and with the present protocol we aimed to mimic the oscillations in energy stores seen with frequent exercise training. For muscle glycogen (Table 2) this aim was achieved, but the oscillations did not translate into an improvement of insulin-mediated glucose clearance with ADF alone, which is in contrast to earlier findings in young, healthy subjects (Halberg et al., 2005). However, the data are in line with findings in obese people, using a calculated index for insulin sensitivity (S_I_) from an intravenous glucose tolerance test (Catenacci et al., 2016) or meal test (Liu et al., 2020), and in non-obese healthy humans using indices derived from oral glucose tolerance tests (Stekovic et al., 2020), even though zero calorie intake on fasting days was only used in one (Liu et al., 2020) of these studies. However, in the present study, the additional weight loss (ADF + WL) resulted in an improvement in insulin-mediated glucose uptake (Table 1).

Insulin action at the hepatic level, i.e. inhibition of endogenous glucose Ra, did not change with ADF. This finding is in line with the lack of effect of ADF on peripheral insulin action. It may require an extended period of starvation (3–4 days) before a reduction of insulin-induced suppression of hepatic glucose output is seen (Fery et al., 1990), but this is not always the case (Vendelbo et al., 2012). Biomarkers for hepatic function (ASAT and ALAT) were seen to decrease with ADF + WL, along with a decrease in intrahepatic triglyceride content. This indicates a general improvement in hepatic function elicited by dietary regimen.

The lack of increases in insulin sensitivity with ADF is in line with the general lack of increases in proteins relevant for skeletal muscle insulin action, e.g. GLUT4, hexokinase, glycogen synthase (Supplementary Figure S1). In rodents, a similar lack of change in hexokinase after intermittent fasting has been reported (Real-Hohn et al., 2018). The amount of intramyocellular lipids is inversely correlated with insulin sensitivity (Pan et al., 1997; Hansen et al., 2016), and the lack of changes in lipid droplets (Figure 5) and triglyceride content in m. psoas major fit well with the lack of changes in insulin sensitivity. A similar amount of lipid content in the muscle in T2DM and OB has been shown before (Hansen et al., 2016) and in the present study intramuscular triglyceride in the biopsies from the leg (vastus lateralis) were also similar between the study groups and did not change with the intervention.

Plasma concentrations of triglycerides decreased markedly (up to 43%) with ADF and ADF + WL, but not with 30 h of fasting (Table 2). The latter is in line with earlier findings that demonstrated that it requires prolonged fasting (e.g. 28 days (Streja et al., 1977)) before even a small reduction in plasma triglyceride is seen. Lipolysis increases at the beginning of a fasting period (here evidenced by increased FFA and glycerol after 30 h fasting; Table 2). 30%–40% of the FFA is re-esterified in short-term fasting (<2 days), which explains why the triglyceride concentration in plasma at 30 h fasting is not decreased. However, it is puzzling that triglyceride concentrations decrease more with a dietary pattern of “fast” and “feast” than with prolonged fasting itself (Streja et al., 1977). The more so, because the anti-lipolytic effect of insulin diminishes with fasting (Jensen et al., 1987), albeit this is found with a longer duration of fasting than in the present study. Support for an ADF-induced triglyceride-lowering effect is found in low-calorie refeeding studies that demonstrated increased triglyceride turnover and removal efficiency (Streja et al., 1977). The magnitude of plasma triglyceride reduction with ADF and ADF + WL was comparable to reductions found after a major weight loss induced by gastric bypass after a 2 years follow-up (Carbajo et al., 2017) and with a weight loss of more than 10%–15% by lifestyle intervention in obese patients with type 2 diabetes (Wing et al., 2011).

During the fasting days in the present ADF protocol, it would be reasonable to assume that a large part of the substrates for energy production comes from lipids. If not from intramuscular stores, of which a decrease could not be detected, then from extramyocellular stores, i.e. adipose tissue. The amount of adipose tissue decreased (Table 1) during the 6-week intervention, which in turn give rise to the increased availability of fatty acids (Table 2) that facilitates an increased fatty acid transport across the sarcolemma. To this end, we measured fatty acid translocase (CD36), fatty acids transport protein 4 (FATP4), and plasma membrane fatty acid binding protein (FABPpm) which are important players in the transport of fatty acids across the plasma membrane. A mixed result was seen, with CD36 increasing significantly in the OB group, FATP4 decreasing in T2DM, and decreasing in FABPpm (main effect) (Supplementary Figure S1). The changes were small, and the data cannot support the notion that fatty acid transport was increased. Most likely, the oscillation of carbohydrate and lipid substrates every other day blurred a potential marked increase in these proteins. Once inside the muscle cell, fatty acids can be stored as triglycerides and the final step in the synthesis is catalyzed by diglyceride acyltransferase 1 (DGAT1). DGAT1 protein expression did not change with the intervention (Supplementary Figure S2M), but even though we did not detect a difference in lipid content between the two groups, DGAT1 was significantly higher expressed in T2DM compared with OB. To our knowledge, DGAT1 protein expression in skeletal muscle of patients with type 2 diabetes has only been measured in one other study, in which no change was found compared with obese people and athletes (Bergman et al., 2018). Our data suggest that T2DM have the capacity to synthesize greater amounts of intramuscular lipids.

We measured two proteins involved in lipolysis (adipose triglyceride lipase (ATGL), monoacylglyceride lipase (MGLL)) and in lipid storage (Perilipin 2 (adipophilin), perilipin 3 (TIP47), and perilipin 5 (OXPAT)) and in line with the unchanged lipid content in the muscles (Figure 5) we found no effect of the intervention on these proteins (Supplementary Figure S1). An increase in medium-chain acyl-CoA dehydrogenase (MCAD) might have been seen because MCAD is involved in medium-chain fatty acid beta-oxidation, which would be expected to increase with increased fatty acid availability but not with increased lipid storage. However, no change was detected (Supplementary Figure S1). The expression of proteins involved in lipid transport, synthesis and storage presented here, are in line with data on gene expression (mRNA) of many of these proteins in a study on females undergoing an intermittent fasting regimen with the muscle biopsies obtained in the same condition (i.e. after an overnight 12-h fast) (Liu et al., 2020). However, in that study (Liu et al., 2020), a decrease in fatty acid translocase (CD36) mRNA levels was reported, while we found an increase in the protein expression (Supplementary Figure S1). Others have also found that CD36 mRNA remains unchanged with a zero-calorie ADF regimen (Heilbronn et al., 2005).

Compared with minor caloric restriction, ADF over 6 months does not bring about superior health benefits in terms of body weight, body composition, or cardiovascular risk factors in patients with obesity (Trepanowski et al., 2017). While that study also was designed with alternating “fast” and “feast” days as in the present study, there was a built-in caloric restriction of on average 25% (25% of energy needs were allowed as lunch on fast days but this energy deficit was only compensated by a 125% energy intake on feast days) (Trepanowski et al., 2017). This difference in design as well as differences in study cohorts between the two studies makes a direct comparison difficult. The present study was more aggressive by employing zero calorie intake on fast days, which was almost fully compensated on feast days (body weight was used as a biomarker for daily caloric intake, and that decreased only ∼1% during ADF). The second part of the present study, where *ad libitum* diet was allowed on feast days demonstrated that the study participants did not inadvertently compensate the overall caloric deficit, because body weight decreased faster in the latter part of the study. If weight loss is the purpose of ADF, zero-calorie intake must therefore be recommended on fast days because it will not be compensated on feast days. The length of the fasting may also play a role. In the study by Trepanowski et al. (Trepanowski et al., 2017), this was 22 h (K.A. Varady, personal communication), but since lunch was allowed between 12:00 and 14:00 on fasting days, the fasting period was, in fact, two periods of 12 and 10 h. These relatively short periods of fasting every other day may therefore be the reason that this intervention was not superior in reducing body weight compared to ordinary everyday caloric restriction. In the present study, each zero-calorie fasting period was 30 h, which is of sufficient length to markedly draw from endogenous energy sources, introducing loss of body weight and also mimicking oscillations in energy stores induced by exercise (Dela et al., 2019).

In line with previous studies (for review see Dela et al. (Dela and Helge, 2013)) and newer studies (Lund et al., 2018; Dela et al., 2019) we confirm that insulin resistance (Table 1) is not associated with skeletal muscle mitochondrial respiratory function (Supplementary Figure S1). In the present study, we tested ADP sensitivity of the skeletal muscle mitochondria (Supplementary Figure S1) but found no difference between the groups or an effect of ADF. Previously, in patients with type 2 diabetes, we have demonstrated increased sensitivity for complex I (glutamate) and complex II (succinate) substrates (Larsen et al., 2011), but to our knowledge, ADP sensitivity has not previously been measured in this population.

This study has some limitations. We did not randomize patients to a non-intervention control group, because it is a well-known risk that patients assigned to passive control groups may exhibit behavioural changes, especially in studies with a focus on dietary behaviour. Instead, we performed two baseline experiments that were carried out two to 3 weeks apart (Figure 1) to account for any variation in methodology and to avoid a time effect of enrollment into a dietary study *per se*. We did not include a group that performed conventional caloric restriction, thus we cannot make a direct comparison between ADF and conventional caloric restriction, and this was not the purpose here. The last part of the intervention (ADF + WL) was exploratory, thus it should be notet that it cannot entirely be excluded that the increase in glucose clearance rates and insulin secretory response with ADF + WL was the result of additional 3 weeks of ADF, implying a carry-over effect.

The intervention was well tolerated by all patients. The strict zero-calorie regimen is a quite demanding approach, but the reports from the participants were that the most difficult task was to eat the double diet on non-fasting days during the first 3 weeks. However, a double diet every other day was only used for mechanistic reasons, and it is not the recommended approach for the general use of ADF. It should also be noted that oral medication, except antihypertensive drugs but including glucose lowering drugs, was discontinued during the entire intervention. Yet, the patients with type 2 diabetes experienced an improvement in fasting glucose and even HbA1c. This suggests that shorter term (6 weeks) ADF is a feasible approach in patients in treatment with oral glucose-lowering therapy that will bring about loss of weight and improved glycemic control. Longer-term (more than 6 weeks) effects (i.e. physiological and/or psychological) of strict ADF should be assessed before a general recommendation can be given.

## Data Availability

The raw data supporting the conclusions of this article will be made available by the authors, without undue reservation.
