# Supplementary material for: Metabolic effects of alternate-day fasting in males with obesity with or without type 2 diabetes
Source: Front Physiol. 2022 Dec 1;13:1061063. doi: 10.3389/fphys.2022.1061063 (PMC9748572; doi:10.3389/fphys.2022.1061063)
Supplement: Supplementary file 1 [file DataSheet1.DOCX]

# Supplementary material.

## Western Blotting

Protein content was examined using SDS-PAGE (Sodium Dodecyl Sulphate Polyacrylamide Gel Electrophoresis) and Western blotting techniques. First, homogenates were prepared from the samples and the loading solution was mixed. Secondly, the proteins were separated according to their molecular weight by SDS-PAGE. Thirdly, UV irradiation activated the trihalo-compounds in the gel and the proteins were transferred to a polyvinyldiflorid (PVDF) membrane. Fourth, by the use of specific antibodies, the proteins of interest were detected and visualized. Enhanced chemiluminescence Western blotting detection system (ECL, GE Healthcare, Little Chalfont, UK) was used for visualization. The highly sensitive luminol-based ECL-solution was mixed 1:1 and applied to the membrane. The membrane was placed in a transparent plastic cover and placed in a CCD camera (LAS 4000, GE Healthcare, Little Chalfont, UK). Quantification was performed using the ImageQuant TL software (version 7, GE Healthcare). All protein bands on the image from the ECL-activated PVDF membrane were marked and the background was deducted. The total loaded protein content for each sample was quantified from the UV image and the background was deducted. Each sample was normalized to the total protein content (from the UV image). To equalize any membrane-specific differences each sample was calibrated to the mean of the human internal standard samples from the corresponding membrane. All samples were set relative to the mean of the baseline samples from the OB group.

Details on the used antibodies and representative band are shown in Table S1.

Table S1: Antibodies used for Western blotting.

| **Protein** | **Size**  **[kDa]** | **Primary**  **antibody** | **Secondary**  **antibody** | **Representative bands** |
| --- | --- | --- | --- | --- |
| **GLUT-4**  (Rabbit) | 46 | Fischer Scientific  PA1-1065 | Cell Signaling Technology  7074 | 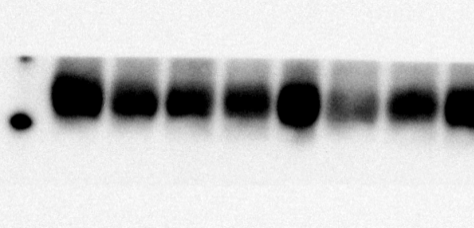 |
| **Hexokinase II**  (Rabbit) | 102 | Cell Signaling Technology  2867 | Agilent Dako  P0448 | 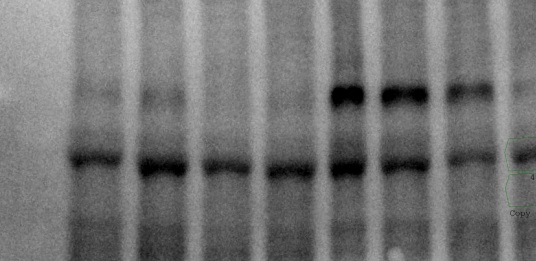 |
| **Pyruvate kinase**  (Rabbit) | 60 | Cell Signaling Technology  3106 | Cell Signaling Technology  7074 | 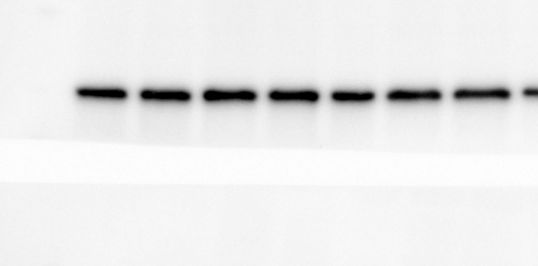 |
| **Glycogen synthase**  (Rabbit) | 84 | Cell Signaling Technology  3893 | Cell Signaling Technology  7074 | 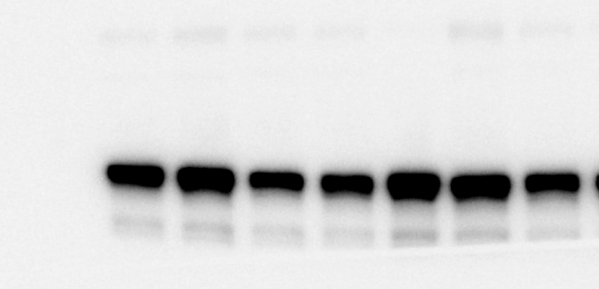 |
| **Glycogen phosforylase**  (Rabbit) | 97 | Agrisera  AS09 | Cell Signaling Technology  7074 | 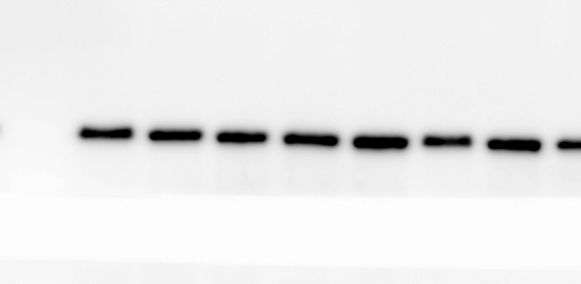 |
| **AKT (pan)**  (Rabbit) | 60 | Cell Signaling Technology  4691S | Cell Signaling Technology  7074 | 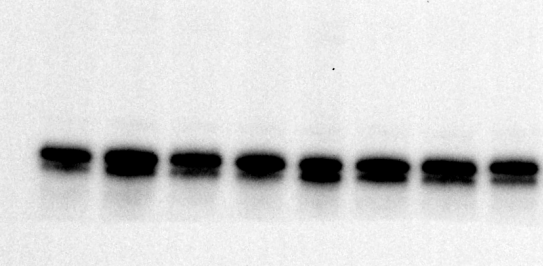 |
| **AS160**  (Rabbit) | 160 | Cell Signaling Technology  2670 | Agilent Dako  P0448 | 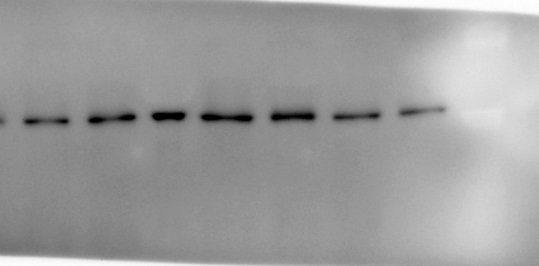 |
| **AMPKα1**  (Rabbit) | 62 | Cell Signaling Technology  5832 | Cell Signaling Technology  7074 | 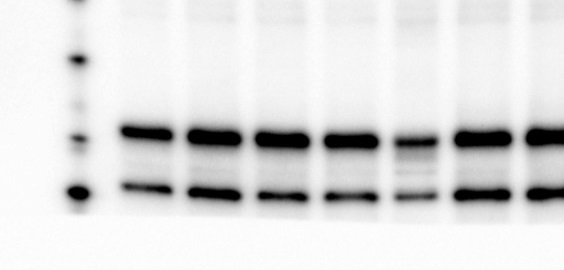 |
| **PKCθ**  (rabbit) | 80 | Abcam  110728 | Cell Signaling Technology  7074 | 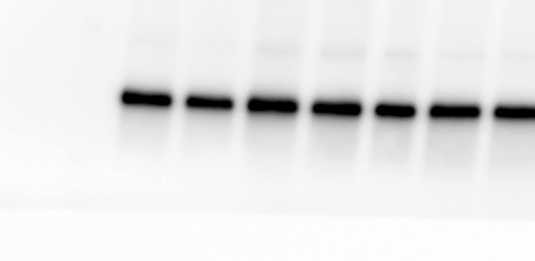 |
| **CD36**  (Goat) | 85-90 | R&D Systems  AF1955 | Agilent Dako  P0448 |  |
| **FATP4**  (Rabbit) | 72 | Abcam  Ab200353 | Cell Signaling Technology  7074S |  |
| **FABPpm**  (Rabbit) | 40 | Abcam  Ab180162 | Cell Signaling Technology  7074S |  |
| **DGAT1**  (Rabbit) | 55 | Abcam  Ab181180 | Cell Signaling Technology  7074S |  |
| **ATGL**  (Rabbit) | 54 | Cayman Biochemicals  #10006409 | Cell Signaling Technology  7074S |  |
| **MGLL**  (Rabbit) | 33 | Abcam  Ab24701 | Cell Signaling Technology  7074S |  |
| **PLIN2**  (Guinea pig) | 52 | PROGEN  GP40 | Abcam  Ab6908 |  |
| **PLIN3**  (Rabbit) | 47 | Sigma-Aldrich  HPA006427 | Cell Signaling Technology  7074S |  |
| **PLIN5**  (Rabbit) | 50 | Novus Biologicals  NB110-60509 | Cell Signaling Technology  7074S |  |

The band are shown in the following order: OB baseline – OB ADF – T2DM baseline – T2DM ADF.

Abbreviations:

OB, obese patients. T2DM, patients with type 2 diabetes. ADF, alternate-day fasting.

GLUT-4, glucose transport protein 4; Akt (pan), Protein kinase B; AS160, AKT substrate of 160 kDa; AMPKα1, 5’ adenosine monophosphate-activated protein kinase; PKCθ, protein kinase C theta; CD36, Cluster of differentiation 36 (fatty acid translocase); FATP4, fatty acid transport protein 4; FABPpm, plasma membrane-associated fatty acid-binding protein; DGAT1, diglyceride acyltransferase 1; ATGL, adipose triglyceride lipase; MGLL, monoacylglycerol lipase; PLIN2, perilipin 2 (adipophilin / adipose differentiation-related protein (ADRP)); PLIN3, perilipin 3 (tail-interacting protein of 47 kD (TIP47)); PLIN5, perilipin 5 (OXPAT).

**Figure S1**

Legend Figure S1: Protein expression (by Western blot) of proteins in skeletal muscle biopsies obtained at baseline and after alternate day fasting (ADF). Individual values are shown. Representative chemiluminescence blots of all proteins are shown in the supplementary material. The difference between groups and the possible effect of the intervention was tested by mixed models method with appropriate post hoc analysis if significant interaction (time x group) was seen. a) Glucose transporter protein 4 (GLUT4); b) Hexokinase II; c) Pyruvate kinase; d) Glycogen synthase; e) Glycogen phosphorylase; f) Akt (Protein kinase B); g) Akt substrate of 160 kDa (AS160); h) 5' AMP-activated protein kinase (AMPKα); i) Protein kinase C zeta (Protein kinase C ζ); j) Fatty acid translocase (CD36); k) Fatty acid transport protein 4; l) Fatty acid binding protein plasma membrane; m) Diacylglycerol O-acyltransferase 1 (DGAT 1); n) Adipose triglyceride lipase (ATGL); o) Monoacyl glycerol lipase; p) Perilipin 2 (PLIN2); q) Perilipin 3 (PLIN3); r) Perilipin 5 (PLIN5); s) Medium-chain acyl-CoA dehydrogenase (MCAD).

**Mitochondrial respiratory capacity protocol**

The following protocol was applied in duplicate at 37 degrees Celcius after hyperoxygenation. Malate (2 mM) and glutamate (10 mM) were added to obtain leak respiration (state 2), this was followed by titrating ADP in the following concentrations (0.025 – 0.05 – 0.1 – 0.25 – 0.5 – 1.0 – 2.5 – 5.0 mM) to access complex I linked respiratory capacity (state 3). This was followed by the addition of cytochrome c (10 µM), to test the integrity of the outer mitochondrial membrane. Then succinate (10 mM) was added for maximal complex I + II linked respiratory capacity and finally, cyanide-p-trifluoromethoxyphenylhydrazone (FCCP) was titrated in steps (0.25 µM; until respiration decreased) to measure electron transport system (ETS) capacity. No increase was observed after adding cytochrome c indicating that the outer mitochondrial membrane was intact.

H_2_O_2_ emission (reactive oxygen species (ROS) production) protocol:

The following protocols were applied in duplicate at 37 degrees Celcius in 500 µl quartz cuvettes. Amplex Red (50 µM), horseradish peroxidase (5U/ml), superoxide dismutase (5KU/ml) and blebbistatin (25 µM) were added to 485 µl of respiration medium, and then 2 different protocols were applied. 1: Malate (2 mM) and pyruvate (5 mM) were added, followed by succinate (3 mM); 2: Malate (2 mM) and palmitoyl carnitine (25 µM).

**Figure S 2**

Legend Figure S 2. Skeletal muscle mitochondrial function at baseline and after 3 weeks of alternate day fasting (ADF). a) Respiration expressed per mitochondrial mass (CS activity). Respiration in the buffer medium (routine (Rou)), addition of malate (state 2 respiration complex I (M2)), addition of glutamate (state 2 respiration complex I (GM2)), stepwise addition of increasing concentrations of ADP (state 3 respiration complex I), addition of cytochrome c (quality control (CytC)), addition of succinate (state 3 respiration with dual electron input complex I+II (GMS3)), and finally addition of protonophore (uncoupled respiration (FCCP)). b) Reactive oxygen species (measured as H_2_O_2_ flux) with addition of palmitoyl carnitine (Palm car), addition of stepwise increasing concentrations of succinate. c) ADP sensitivity (apparent Km). d) Maximal oxidative phosphorylation capacity (OXPHOS) (Vmax). Values are mean ± SD (a and b) or individual values (c and d).

***IMTG quantification***

Muscle cryosectioning - A Leica CM3050 S Cryostat was used to cut 16 µm muscle cryosections. The orientation of the object holder was adjusted for the Cryostat to cut perpendicular to the muscle fibres, to achieve cross-sectional sections (Figure S1 B) and avoid longitudinal sections (Figure S1 A).

**A**

**B**


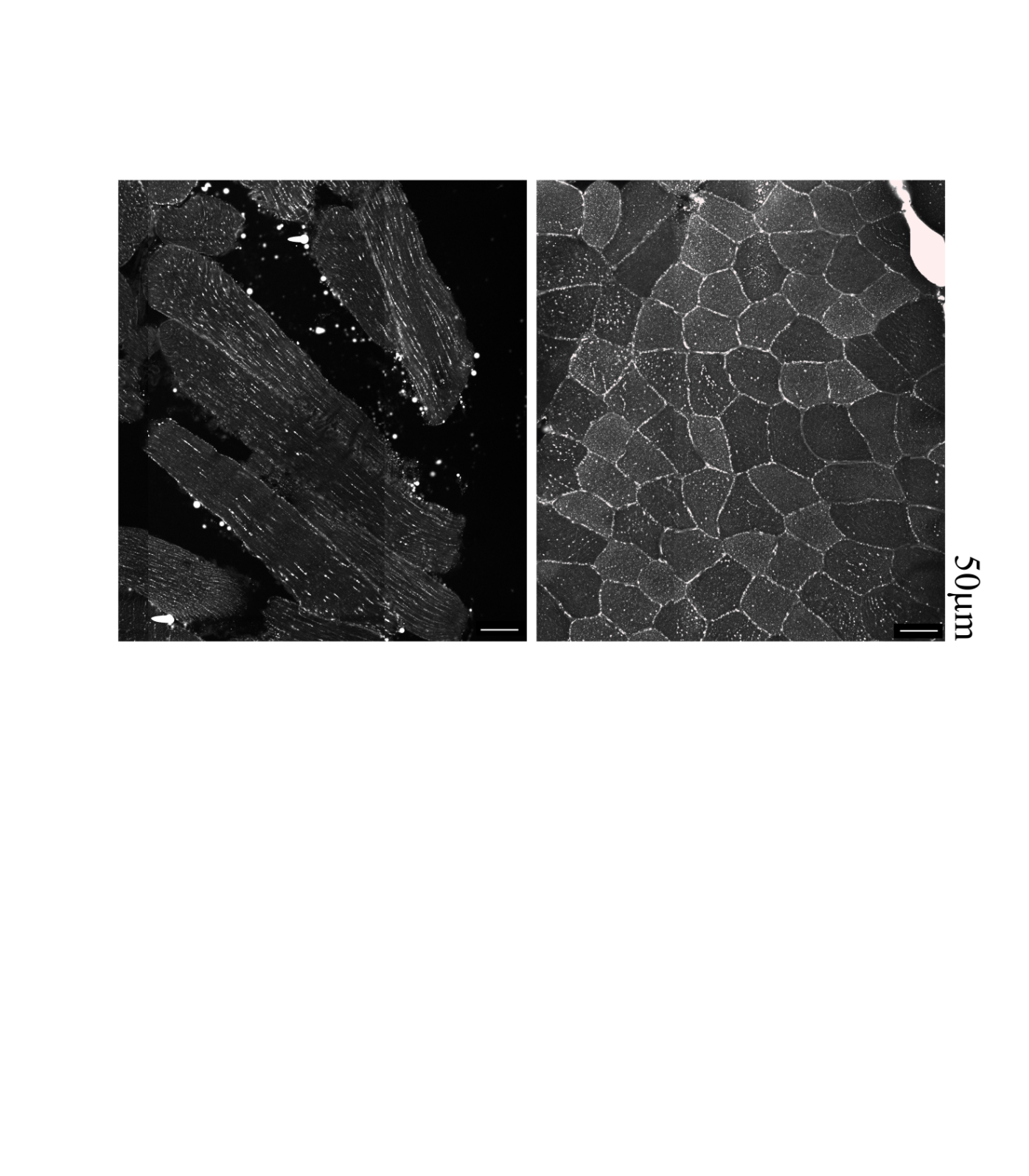


Figure S3 Cryosection cutting. Depending on how the object holder is orientated, the cryosections will be cut longitudinal (A) or perpendicular (B) to the muscle fibres. Scale bar 50 µm.

The cryosections were mounted onto Superfrost+ glass slides, with two consecutive cryosections on each. The cryosections were right after mounting rapidly fixed in 4% Zamboni fixative [4% depolymerized paraformaldehyde supplemented with 0.15% picric acid ] (Region H Pharmacy) for 30 minutes.

Bodipy staining - The slides were then washed 2x 5 minutes in PBS [1.4 mM KH_2_PO_4_, 8mM Na_2_HPO_4_, 2H_2_O, 140 mM NaCl, 2.7 mM KCl and MilliQ water adjusted with HCl to pH 7.3] and incubated with Bodipy-493/503 (20 µg/ml PBS) for 30 minutes. Bodipy is sensitive to light and the slides were therefore kept in the dark throughout the remaining steps. The slides were then washed 3x 10 minutes in PBS before they were mounted with Vectashield Mounting Medium (H-1000 from Vector Laboratories) and covered with a glass coverslip #1.5.

*Imaging and Quantification*

Image acquisition was performed with a Zeiss LSM700 confocal microscope, through a Plan-Apochromat 20x/0.8 objective (pixel size 0.20 µm^2^). The 488 nm argon laser was used for excitation of the Bodipy-493/503 and, IMTG staining was collected in the range 541-634 nm.

**A**

**B**

**C**


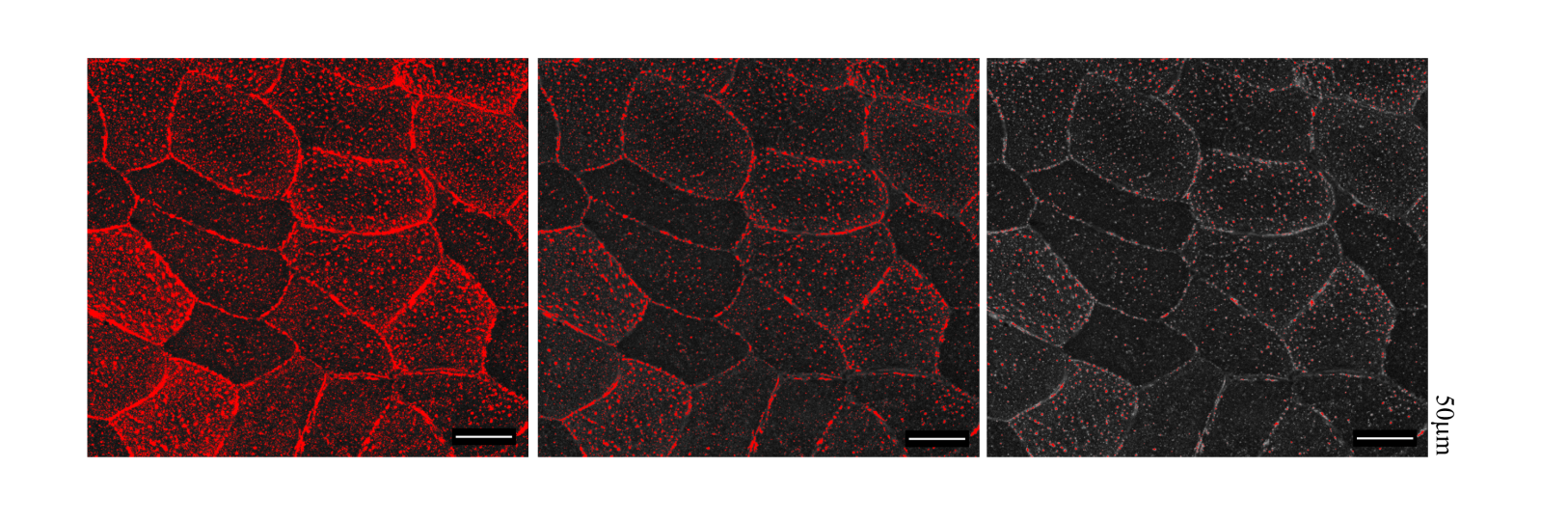


Figure S4 Visual assessment of threshold. Representative images of the threshold being set too low (A), at an appropriate level (B) and too high (C). Scale bar 50 µm.

Using Fiji software (1) muscle fibres were manually defined and added to the ROI manager (178 ± 83 muscle fibres per biopsy). The threshold for IMTG staining was set individually for each staining condition, to include small lipid droplet signals, but to avoid subsarcolemmal lipid staining (1) (Figure S illustrates representative images of the threshold being set too low (A), appropriately (B), and too high (C)). The measurement of fractional area and lipid droplet sizes were obtained for each cell.

***Recruitment***

**Figure S5**


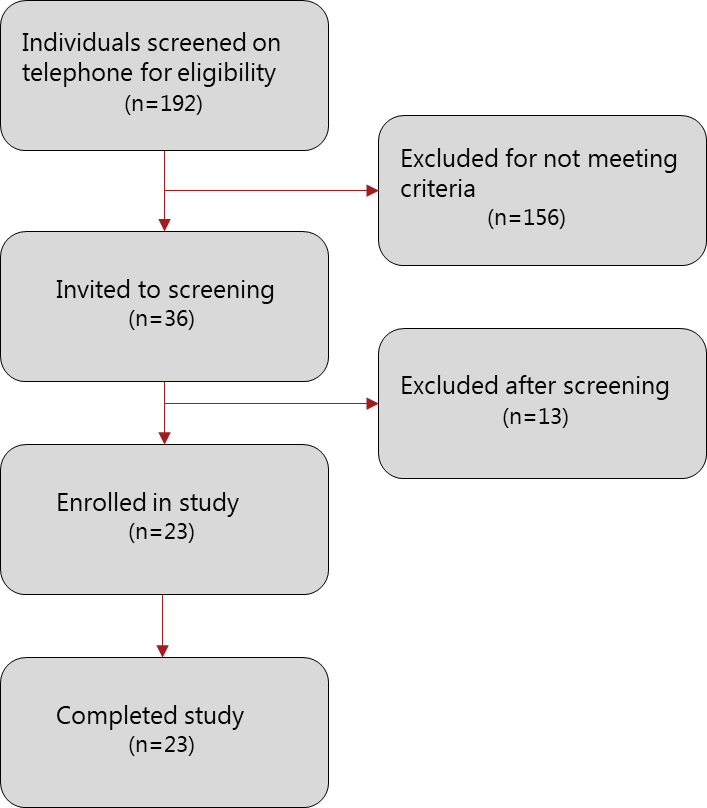


Reference List

1. Schindelin J, Arganda-Carreras I, Frise E, Kaynig V, Longair M, Pietzsch T, Preibisch S, Rueden C, Saalfeld S, Schmid B, Tinevez JY, White DJ, Hartenstein V, Eliceiri K, Tomancak P, Cardona A: Fiji: an open-source platform for biological-image analysis. *Nat Methods* 2012;9:676-682.
